# Intersectional Discrimination Attributions and Health Outcome among American Older Adults

**DOI:** 10.1093/geroni/igaa057.3222

**Published:** 2020-12-16

**Authors:** Peiyi Lu, Dexia Kong, Mack Shelley, Joan Davitt

**Affiliations:** 1 Iowa State University, Ames, Iowa, United States; 2 Rutgers University, New Brunswick, New Jersey, United States; 3 University of Maryland, Baltimore, Baltimore, Maryland, United States

## Abstract

Discrimination has been consistently documented to relate to adverse health outcomes. However, most existing research focused on a single discrimination attribution (e.g. ageism). Few studies considered multifaceted discrimination attributions. Guided by an intersectionality framework, this study examined intersectional discrimination attributions and their associations with health outcomes. Older respondents (aged >50) from the Health and Retirement Study in 2014-2015 were included in the analysis (n=6,286). Their experiences and self-perceived reasons (age, gender, sexual orientation, race, national origin, religion, financial status, weight, physical appearance, disability, and others) for everyday discrimination were examined. Latent class analysis was employed to ascertain the profiles of subgroups characterized by their intersectional discrimination attributions. Regression models examined the correlates of the class memberships and the associations with health outcomes. Six classes were identified: class 1 (54.52% of the sample) had no/minimal discrimination experience; Class 2 (21.89%) experienced primarily ageism; class 3 (8.81%) reported discrimination based on age/gender/national origin/race; class 4 (7.99%) attributed discrimination to financial/other reasons; class 5 (5.87%) experienced discrimination based on age/weight/physical appearance/disability; and class 6 (0.92%) perceived discrimination from almost every aspect. Intersectional discrimination attributions were associated with poorer self-rated health, and greater levels of depressive symptoms and loneliness. The associations between intersectional discrimination and cognition were not statistically significant. This study found multiple marginalized identities co-occur and compound to contribute to perceived everyday discrimination among American older adults. Those experiencing discrimination due to multiple reasons warrant particular attention. Results underscore the utility of an intersectional approach in understanding discrimination in later life.

